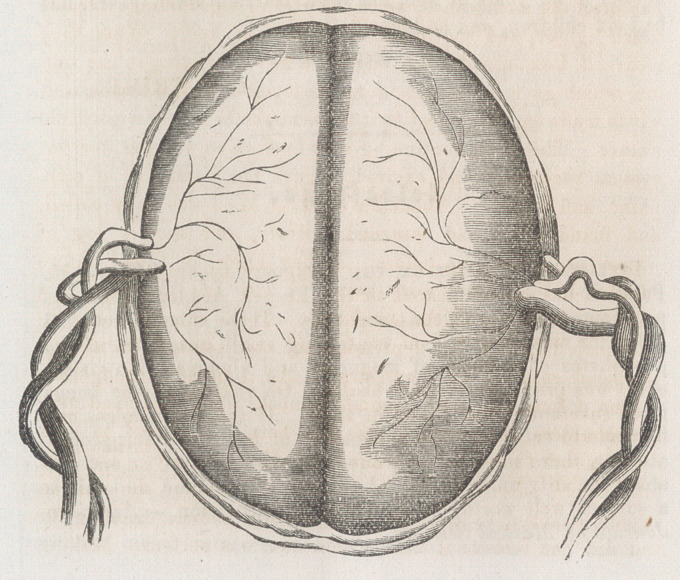# Letter from D. B. Trimble, M.D.

**Published:** 1871-03

**Authors:** D. B. Trimble

**Affiliations:** Chicago


CHICAGO, *February 15, 1871.*

DR. N. S. DAVIS,—DEAR SIR,—The following case, from its rarity, may be of some interest to your readers, and merit a place in THE EXAMINER:

On Sunday, the 12th of February, I was called about four o'clock P.M., to attend Mrs. R. in her fifth confinement; it was premature, as I had been engaged for the 15th of April. Her previous labors had all been very rapid and easy. She was taken in labor about 12 o'clock, M., and when I arrived I found her pains frequent and quite severe, though she was sitting up. At the first examination, found the vagina relaxed and moist, but could not reach the os uteri. She was unusually restless and irritable, and soon insisted on getting out of bed, but had been on her feet only a few moments, when she exclaimed that the child was coming, and on examining found the bag of waters projecting from the vulva. Laying her down, two more pains ruptured the membranes, when the uterine contractions became much lighter, and continued so for half an hour. When they returned, I examined again, and felt a soft body lying in the vagina, which gave the sensation to the touch of a firm tumor, and which made me apprehend that there might be one of a polypoid nature. The pains continuing very ineffective, and her nervous system becoming much excited, I gave her a solution of morphine, and left her. About 2 o'clock, A.M., I was again called, and found labor had returned actively, and *another* bag of waters protruding, as before. I then knew, as I had before suspected, that there were twins. I now felt the face of a child presenting, with the occiput towards the sacrum, but without much difficulty, (by depressing the chin towards the chest), the head entered the lower strait. As it passed through the vagina, I found the cord around the neck, which I slipped over the shoulders, and the child was soon born. It proved to be a seven month fœtus, but was alive and cried strongly. The umbilical cord pulsating with unusual force, I allowed the child to remain attached to it, at least ten minutes before removing it; and did not remove it until respiration was perfect. Making gentle traction on the cord, and finding it unyielding, and the patient having no pain, I desisted from interference, until the return of the pains in about half an hour, when, with slight assistance, I brought the placenta away entire; but found that I could not detach it, and upon examination perceived the other cord was attached to it. I enclosed it in a towel and let it remain, her pains again subsiding.

Having now full knowledge of the case, and knowing that unless there was a very speedy delivery, the other child must perish, I tried, by manipnlating the abdomen, to induce a return of the uterine contractions, and, after a time, succeeded.

The second child presented the head in the second position, the vertex towards the symphisis pubis, preceeded by the arm. With considerable difficulty I returned the arm by rotating it over the head and face, when delivery was soon accomplished. The cord was, with this child, around the neck and abdomen. The child was dead. The first was born at 4 o'clock, the second at 5 o'clock, A.M. On examining the placenta, I found it about the size of an ordinary placenta at full term; with a sulcus in its long diameter (it was somewhat oblong), and presenting on its fœtal surface two hemispheres, bearing considerable resemblance to those of the brain. At right angles to the sulcus, and at the edge of the placenta, one cord was inserted on either side. A very good representation of fœtal face of the placenta is presented on the preceding page.

The double set of membranes, with the single placenta, with the unusal places of insertion of the cords, form a singular departure from ordinary cases, and is, I suppose, a modification of the Battledore placenta. The bloodvessels from the two cords anastamosed in the placenta, which is also an unusual circumstance. In a practice of thirty years I have never seen a similar case; and as these anomalies rarely occur, they should, I think, be recorded, as being at least interesting, if serving no practical purpose. I may observe, in conclusion, that the mother is 23 years of age, has been married seven years, has had six children, and is doing well.

Very respectfully,

D. B. TRIMBLE.

## Figures and Tables

**Figure f1:**